# Protective Role of Ethanol Extract of *Cibotium barometz* (Cibotium Rhizome) against Dexamethasone-Induced Muscle Atrophy in C2C12 Myotubes

**DOI:** 10.3390/ijms241914798

**Published:** 2023-09-30

**Authors:** Na-Hyung Kim, Joo-Yeon Lee, Choon Young Kim

**Affiliations:** 1 Department of Food and Nutrition, Yeungnam University, Gyeongsan 38541, Gyeongbuk, Republic of Korea; knahyu@yu.ac.kr (N.-H.K.); jooyeonlee@ynu.ac.kr (J.-Y.L.); 2 Institute of Human Ecology, Yeungnam University, Gyeongsan 38541, Gyeongbuk, Republic of Korea

**Keywords:** *Cibotium barometz* (Cibotium Rhizome), sarcopenia, muscle atrophy, regulated in development and DNA damage responses 1, kruppel-like factor 15

## Abstract

Sarcopenia is a progressive muscle disease characterized by the loss of skeletal muscle mass, strength, function, and physical performance. Since the disease code was assigned, attention has been focused on natural products that can protect against muscle atrophy. *Cibotium barometz* (Cibotium Rhizome) has been used as an herbal medicine for the treatment of bone or joint diseases in Asian countries. However, no studies have identified the mechanism of action of Cibotium Rhizome on muscle atrophy related to sarcopenia at the site of myotubes. The aim of this study was to investigate the improvement effect of the ethanol extract of Cibotium Rhizome (ECR) on dexamethasone-induced muscle atrophy in an in vitro cell model, i.e., the C2C12 myotubes. High-performance liquid chromatography was performed to examine the phytochemicals in ECR. Seven peaks in the ECR were identified, corresponding to the following compounds: protocatechuic acid, (+)-catechin hydrate, *p*-coumaric acid, ellagic acid, chlorogenic acid, caffeic acid, and ferulic acid. In atrophy-like conditions induced by 100 μM dexamethasone for 24 h in C2C12, ECR increased the expression of the myosin heavy chain, p-Akt, the p-mammalian target of rapamycin (mTOR), p-p70S6K, and repressed the expression of regulated in development and DNA damage responses 1 (REDD1), kruppel-like factor 15 (KLF 15), muscle atrophy F-box, and muscle-specific RING finger protein-1 in C2C12. In addition, ECR alleviated dexamethasone-induced muscle atrophy by repressing REDD1 and KLF15 transcription in C2C12 myotubes, indicating the need for further studies to provide a scientific basis for the development of useful therapeutic agents using ECR to alleviate the effects of skeletal muscle atrophy or sarcopenia.

## 1. Introduction

Sarcopenia is a muscle disease characterized by loss of skeletal muscle mass, strength, and physical performance [1]. As the older population increases rapidly, the number of patients with sarcopenia is estimated to exceed 500 million worldwide by 2050 [2]. Since 2016, as defined by the International Classification of Diseases, Tenth Revision, Clinical Modification diagnosis code M62.84, many studies have focused on the mechanisms of sarcopenia related to skeletal muscle, and its cellular and molecular mechanisms were elucidated due to a decrease in the number and size of skeletal muscle fibers by sophisticated processes including uneven protein homeostasis, mitochondrial dysfunction, a low number of satellite cells, stiffness of the extracellular matrix surrounding skeletal muscle or impaired force transmission, imbalanced autophagy or apoptosis, and elevation of inflammatory cytokines by oxidative stress [3,4].

Among the pathological mechanisms of sarcopenia, atrophy of the skeletal muscle fibers, which can determine muscle mass, is induced by the disruption of protein homeostasis, which regulates the balance between protein synthesis and degradation. The representative pathway of protein synthesis in skeletal muscle is mediated by insulin or insulin like growth factor (IGF)-1 via phosphatidyl-inositol-3-kinase and Akt/mammalian target of rapamycin complex (mTORC) 1 signaling and Forkhead box protein O (FoxO) suppression [5,6,7]. By phosphorylating p70S6 Kinase 1(S6K1) and eukaryotic translation initiation factor 4E (eIF4E) binding protein (4E-BP) 1, which are two key downstream targets of mTOR, mTORC 1 promotes protein synthesis [8,9]. mTOR is a serine/threonine-specific protein kinase that is involved in cell growth and proliferation [10,11]. 

A previous study reported that glucocorticoids markedly suppress protein synthesis rates in skeletal muscle [8]. Recognition of glucocorticoids by glucocorticoid receptors (GRs) induces a conformational change in the receptor that exposes a nuclear localization signal; thereafter, GR binds to the nuclear response elements on the promoters of target genes to activate or inhibit transcriptional factor expression [12,13,14]. The administration of dexamethasone (DEX), a synthetic glucocorticoid, induces the dephosphorylation of 4E-BP1, which strengthens its affinity for eIF4E, competitively inhibits eIF4G-eIF4E coupling, and ultimately prevents assembly of the functional eIF4F homocomplex [8]. Together with the stimulation of skeletal muscle atrophy by promoting protein breakdown through the ubiquitin–proteasome pathways, it has been reported that DEX inhibits protein synthesis by utilizing the Akt/mTOR/S6K1 axis [12,13,15]. Muscle atrophy F-box (MAFbx) and muscle-specific RING finger protein-1 (MuRF1), two muscle-specific E3 ligases associated with ubiquitin-proteasomal degradation in muscle atrophy, are known as muscle proteolysis markers [15]. In addition, MAFbx and MuRF1 are transcriptionally activated by kruppel-like factor 15 (KLF 15), FoxO1, and FoxO3a against DEX [6,16,17]. Moreover, an increased expression of regulated in development and DNA damage responses 1 (REDD1) (also known as DNA damage-inducible transcript 4 (DDIT4)) and KLF15 inhibits mTORC1 activity [18,19].

The *Cibotium barometz* (L.) J. Sm. is a perennial herb belonging to the Cibotiaceae family and the rhizome of it is also named *Cibotii Rhizoma* or *Cibot Rhizoma*. The *Cibotium barometz* (L.) J. Sm. has been reported as a tropical herb that grows naturally on the slopes and valleys of sunny mountains in the highlands of Vietnam, southern China, southern Japan, Taiwan, and Indonesia, and the root of *Cibotium barometz* (L.) J. Sm. is used as a folk remedy for the treatment of bone or joint diseases [20]. Recently, Chinese herbal medicines, including *Cibotii Rhizoma*, have been found to be effective in strengthening muscles and bones, tonifying the liver and kidney, and relieving pain, and an expert consensus has been reached on its use in clinical practice [21]. For instance, applying an ethanol extract of *Cibotium barometz* (Cibotium Rhizome) to neuroblastoma SK-N-SH cells induces neurite recovery and regeneration of nerve cells by reducing Nogo-A expression [20]. However, no reports have revealed the mechanism of the therapeutic effect of Cibotium Rhizome at the muscle cell level, although it has been traditionally used in the treatment of diseases related to bone, joint, and muscle strengthening in Asian countries. To date, no medications for sarcopenia have been approved by the US Food and Drug Administration. Therefore, this study aimed to investigate the improvement effect of the ethanol extract of Cibotium Rhizome (ECR) on DEX-induced muscle atrophy in an in vitro cell model using C2C12 myotubes. In addition, we focused on providing basic data on compounds of ECR, which were analyzed using high performance liquid chromatography, and the expression of transcriptional factors along with the experimental results of this study using a computational tool from public databases that have recently been in the limelight.

## 2. Results

### 2.1. HPLC Analysis

**Figure 1:**
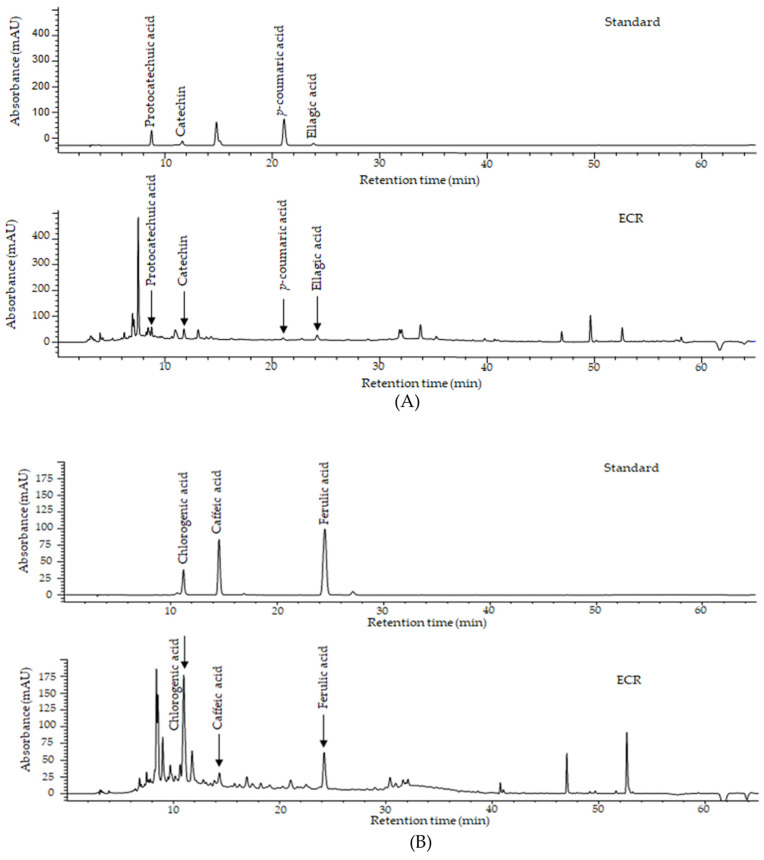
Chromatogram of major compounds of ECR at (**A**) 280 nm and (**B**) 320 nm.

To elucidate the contents of compounds present in the ECR, we performed an HPLC analysis. The chromatograms of the seven compounds are shown in Figure 1. As shown in Table 1, the compounds in ECR were protocatechuic acid, catechin, *p*-coumaric acid, ellagic acid, chlorogenic acid, caffeic acid, and ferulic acid. The amounts of each compound in ECR were 562.28, 2233.07, 166.90, 274.78, 5068.92, 313.30, and 874.05 μg/g, respectively. The structure, classification, and retention time of the peak by chromatogram analysis, values of drug likeness (DL), oral bioavailability (OB), or Caco-2 permeability, and references for each compound in the ECR are presented in Table 1.

### 2.2. Alleviating Effects of ECR in DEX-Induced Muscle Atrophic Cells 

**Figure 2:**
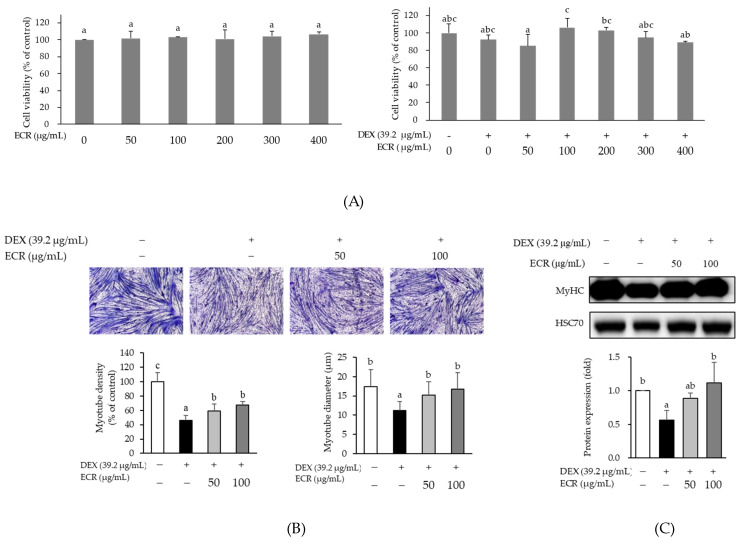

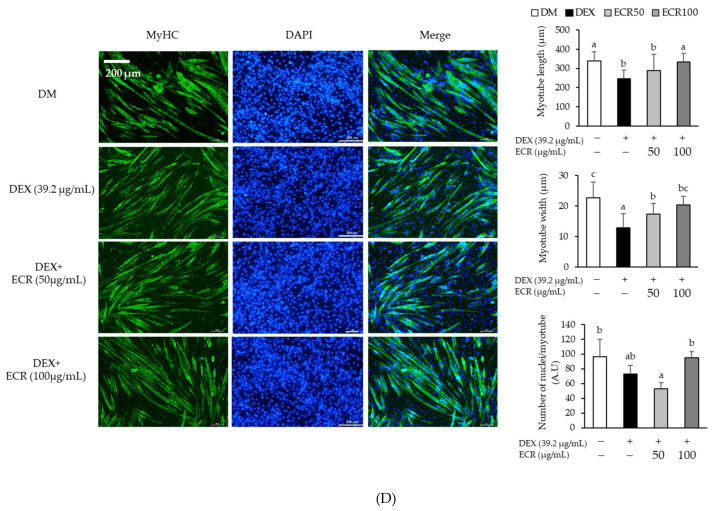
Effects of ECR on (**A**) cell viability, (**B**) myotube density and width, and (**C**) protein expression levels of MyHC. (**D**) Representative fluorescence image of myotube and graphs of myotube density, width, and number of nuclei in C2C12 cells (scale bar, 200 μm). Immunolabeling MyHC antibody (green). Nucleus stained with DAPI (blue). Different letters indicate significant differences by Duncan’s multiple range test (*p* < 0.05).

In the MTT assay, ECR did not affect C2C12 cell viability (Figure 2A). To examine the effect of ECR on DEX-induced myotube atrophy, C2C12 myotubes were treated with DEX (39.2 µg/mL) and ECR (50 and 100 µg/mL) for 24 h. The formation of multinucleated myotubes decreased only in DEX-treated C2C12 myotubes compared with control cells. To observe the morphological changes in DEX-induced myotube, the number of nuclei present in myotubes was calculated. The results of Jenner–Giemsa staining revealed that myotube density and width were increased by ECR treatment (Figure 2B). The DEX-induced downregulation of MyHC protein was also remarkably increased by ECR treatment (Figure 2C). In addition, the length and width of myotubes were significantly increased by ECR treatment. Furthermore, the number of DAPI-stained nuclei localized in MyHC-positive myotubes was calculated. Although the ECR treatment at the concentration of 50 µg/mL tends to reduce the number of nuclei, ECR treatment at the concentration of 100 µg/mL significantly increased the number of nuclei (Figure 2D).

### 2.3. Effects of ECR on Protein Synthesis via the Akt/mTOR/S6K1 Pathway

**Figure 3:**
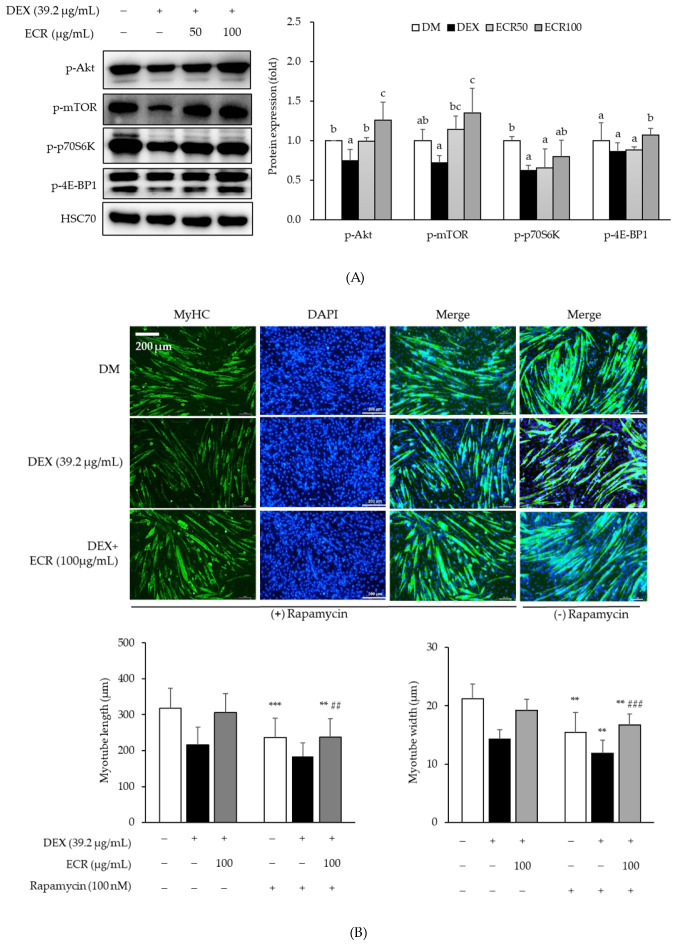
Effects of ECR on (**A**) the protein expression levels of p-Akt, p-mTOR, p-p70s6k, and p-4E-BP1 proteins. (**B**) Representative fluorescence image and myotube density and width by treatment of rapamycin (+/−) in C2C12 cells (scale bar, 200 μm). Immunolabeling MyHC antibody (green). Nucleus stained with DAPI (blue). Different letters indicate significant differences by Duncan’s multiple range test (*p* < 0.05). ** *p* < 0.01 and *** *p* < 0.001 compared with treatment of rapamycin (+/−) in each group. ^##^ *p* < 0.01 and ^###^ *p* < 0.001 vs. DEX treated cells.

To determine the effects of ECR on protein synthesis through the Akt/mTOR/S6K1 signaling pathway, the protein levels of each molecule involved in the pathway were measured in C2C12 myotubes. DEX treatment decreased the phosphorylation of Akt, mTOR, and its downstream substrates, such as p70S6K and 4E-BP1 compared to that in control cells. ECR upregulated the phosphorylation of Akt, mTOR, p70S6K, and 4E-BP1 in DEX-induced muscle atrophy cells compared to that in DEX-treated C2C12 myotubes alone (Figure 3A). In particular, the phosphorylation of Akt and mTOR was upregulated with ECR treatment at the concentration of 100 µg/mL. In addition, we examined the effects of ECR of 100 µg/mL on protein synthesis by treatment with rapamycin, a specific mTORC1 inhibitor, and found that rapamycin treatment decreased myotube length and width compared with non-rapamycin treatment (Figure 3B).

### 2.4. Effects of ECR on Protein Degradation in Myotubes

**Figure 4:**
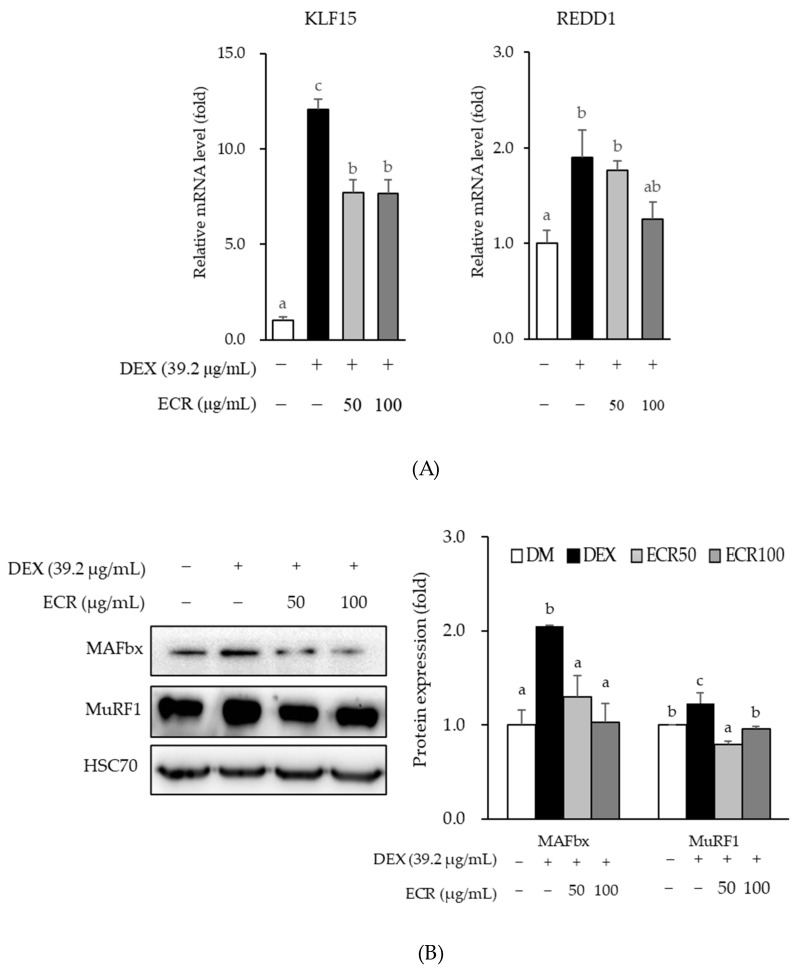
Effects of ECR on (**A**) mRNA expression levels of KLF15 and REDD1, and (**B**) protein expression levels of MAFbx and MuRF1 in C2C12 cells. Different letters indicate significant differences by Duncan’s multiple range test (*p* < 0.05).

In addition to protein synthesis, we investigated the effects of ECR on DEX-induced protein degradation in C2C12 myotubes because an imbalance in protein synthesis and protein degradation induces muscle atrophy. ECR significantly inhibited the mRNA expression of REDD1 and KLF15 and protein expression of MAFbx and MuRF1 in DEX-induced muscle atrophy cells compared with DEX-only treated cells (Figure 4).

### 2.5. Component-Target-Sarcopenia/Muscle Atrophy Pathway Interaction

**Figure 5:**
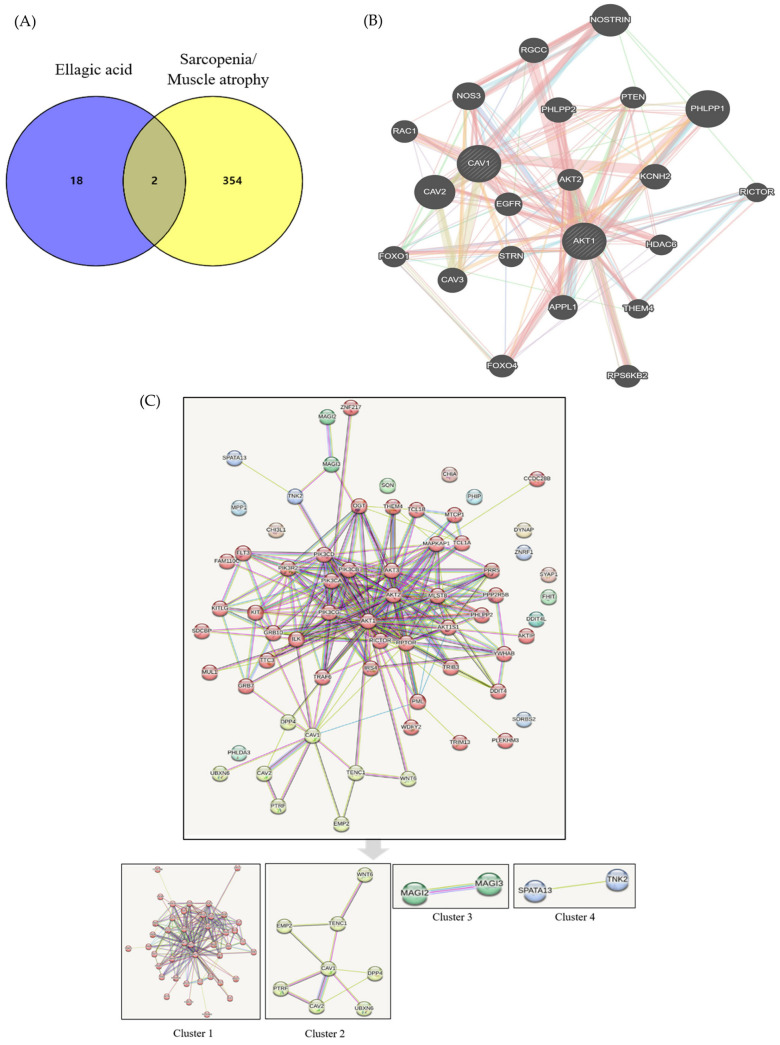
Component–target–disease analysis. (**A**) Venn diagram (**B**) Common target network and (**C**) Four cluster analysis.

Of the three reference values, we selected the compound that fit only two, i.e., the DL and OB values, while omitting the Caco-2 permeability value as seven compounds in ECR confirmed by HPLC did not match the values of DL ≥ 0.18, OB ≥ 30%, and Caco-2 ≥ −0.4 according to absorption, distribution, metabolism, and extraction (Table 1). Ellagic acid, as an active ingredient of ECR, met the above criteria, and its DL and OB values were 0.43 and 43.06%, respectively. We identified 20 targets for ellagic acid and 376 targets excluding pseudogenes for sarcopenia/skeletal muscle atrophy. We also confirmed the presence of overlapped two common elements, AKT1 and caveolin-1 (CAV1). The interaction score was determined to be of medium confidence 0.4. To assess and visualize the interactions between components and targets, we utilized Cytoscape version 3.10.0. The numbers of nodes and edges were 66 and 224, respectively. The average node degree and local clustering coefficient were 6.79 and 0.61, respectively. The *p*-value of protein–protein interaction enrichment was <1.0 × 10^−16^. Targets consisted of four clusters as follows: gene count was 43 and related proteins included AKT1, AKT1S1, AKT2, AKT3, AKTIP, CCDC28B, DDIT4, FAM110C, RLT3, GRB10, GRB7, ILK, IRS4, KIT, KITLG, MAPKAP1, MLST8, MTCP1, MUL1, OGT, PHLPP2, PIK3CA, PIK3CB, PIK3CD, PIK3CG, PIK3R2, PLEKHM3, PML, PPP2R5B, PRR5, RICTOR, RPTOR, SDCBP, TCL1A, TCL1B, THEM4, TRAF6, TRIB3, TRIM13, TTC3, WDFY2, YWHAB, and ZNF217 in red cluster 1; gene count was 8 and related proteins included CAV1, CAV2, DPP4, EMP2, PTRF, TENC1, UBXN6, WNT6 in yellowish green cluster 2; gene count was 2 and related proteins included MAGI2 and MAGI3 in green cluster 3; and gene count was 2 and related proteins included SPATA13 and TNK2 in blue cluster 4 (Figure 5). Of the 245 biological processes, 33 molecular functions, 23 cellular components in GO, and 112 pathways in KEGG were significantly enriched. The top 10 enrichment items on biological processes, molecular functions, and cellular components via the GO and KEGG pathways are shown in Table 2. The “Longevity regulating pathway-multiples species” was predominantly enriched in the KEGG database.

## 3. Discussion

The prevalence of sarcopenia related to muscle atrophy was increased worldwide; however, to date, no medications for sarcopenia have been approved by the US Food and Drug Administration. In this study, we investigated DEX-induced C2C12 myotubes to explore the muscle atrophy-alleviating effects of ethanol extracts of Cibotium Rhizome, which is traditionally used in treatments of diseases related to bone, joint, and muscle strengthening in Asian countries. It was demonstrated that Cibotium Rhizome is effective in strengthening the muscles and bones, tonifying the liver and kidneys, and relieving pain [21]. Previous studies reported skeletal muscle and bone are connected anatomically and physiologically, and play a crucial role in human locomotion and metabolism [28,29]. Myokines derived from myocytes, such as interleukin-6, IGF-1, fibroblast growth factor-2, brain derived growth factor, and myostatin and osteokines derived from bone cells, such as osteoclacin and sclerostin, influence both anabolism and catabolism [28]. The loss of muscle mass and strength can often imply the co-existence of sarcopenia–osteoporosis andis also associated with a loss in bone mass [29]. It is inferred that the effect of Cibotium Rhizome on strengthening bones and muscles [21] is due to this mechanism [28,29]. The active compounds in Cibotium Rhizome have been reported to contain phenolic acids, including protocatechuic acid and caffeic acid [30,31], flavonoids, including kaempferol and onychins [32], and fatty acids, including oleic acid, palmitic acid, and octadecaonic acid [33,34]. Another study demonstrated that Cibotium Rhizome contains compounds such as onitin, onitin-4-O-β-D-glucopyranoside, pterosin R, woodwardinic acid, and tannin [35]. We identified seven compounds in ECR using HPLC: protocatechuic acid, (+)-catechin hydrate, *p*-coumaric acid, ellagic acid, chlorogenic acid, caffeic acid, and ferulic acid.

To investigate the links between identified compounds and sarcopenia or muscle atrophy, we performed an analysis using public computational database tools. Among seven compounds in ECR, ellagic acid was the only compound that matched DL ≥ 0.18 and OB ≥ 30%. Subsequently, AKT1 and CAV1 have been identified in the interactions between ellagic acid and sarcopenia or muscle atrophy. AKT1 is a RAC-alpha serine/threonine-protein kinase that regulates many processes, including metabolism, proliferation, cell survival, growth and angiogenesis [36,37]. AKT1 is an Akt isoform that exhibits extensive homology to the kinase domains of protein kinases A, G, and C [36]. In mammals, AKT1 is composed of an N-terminal pleckstrin homology domain, a central catalytic domain, and a C-terminal hydrophobic domain that is activated by PI3K-dependent or independent signaling pathways [36,37,38]. Our study demonstrated that ECR increased MyHC expression and enhanced protein synthesis through the Akt/mTOR/S6K1 signaling pathway, although we did not investigate the expression of the respective Akt isoforms in myotubes. In the Akt/mTOR/S6K1 signaling pathway, mTOR phosphorylates S6K1, and activated S6K1 promotes translation initiation through activation of the S6 ribosomal protein [8,9]. In addition, mTOR, a serine/threonine-specific protein kinase, promotes translation by phosphorylating 4E-BP1 [8,9,10,11]. Consistent with previous studies using either plant-derived extracts, their major active compounds, or phytochemicals, ECR also increased the width and length of myotubes, the number of nuclei in the myotubes, MyHC expression, and enhanced protein synthesis via the Akt/mTOR/S6K1 pathway in Dex-induced muscle atrophy [39,40,41,42]. Additionally, the action of ECR through the inhibition of mTOR in these pathways was observed after treatment with rapamycin, a specific mTORC1 inhibitor.

An imbalance between decreased anabolic protein synthesis and increased catabolic protein degradation can induce muscle atrophy. Maintaining protein homeostasis is an important preventive or therapeutic approach for muscle atrophy and diseases, such as sarcopenia. In skeletal muscle, there are proteolytic systems, including calcium dependent pathways, caspase dependent pathways, the ubiquitin–proteasomal system, and the autophagy–lysosomal system [43,44]. The active protein degradation systems in muscle are the ubiquitin–proteasomal and the autophagy–lysosomal system [43]. In eukaryotic cells, most proteins in the cytosol and nucleus are degraded via the ubiquitin–proteasome pathway, and proteins destined for degradation by the 26S proteasome are marked by the covalent attachment of ubiquitin chains [45]. Activation of phosphatidylinositol-3-kinase (PI3K)/Akt prevents proteolysis through ubiquitin–proteasomal degradation by inhibiting FoxO [5]. Two muscle specific E3 ligases among E1, E2, and E3 ligases associated with ubiquitin–proteasomal system are MAFbx and MuRF1 [15,46]. Consistent with a previous study, our data showed that MAFbx and MuRF1 protein levels were upregulated by DEX [47]. ECR alleviated the protein expression of MAFbx and MuRF1 increased by DEX in the present study. 

MAFbx and MuRF1 are transcriptionally activated by KLF 15, FoxO1, and FoxO3a in response to DEX [6,16,17]. We also observed that ECR repressed the mRNA expression of KLF15 and REDD1 in DEX-induced muscle atrophy. Glucocorticoid treatment of skeletal muscles has long been known to decrease protein synthesis and increase protein degradation by regulating transcription factor expression [12]. Following glucocorticoid activation and binding to GR, GR translocates to the nucleus and binds to glucocorticoid response elements in the regulatory region, such as promoters, of target genes. These glucocorticoid–GR systems in muscles promote protein degradation through gene sets such as myostatin, atrogin-1, and MuRF1 [6,7]. REDD1, which is activated by glucocorticoids in muscle waste, is also a direct GR target and inhibits mTORC1 by stabilizing the tuberous sclerosis protein 1 (TSC1)–TSC2 inhibitory complex [18,48]. In other words, inhibition of mTORC1 is mediated by a pathway that involves DDIT4/REDD1, AKT1, the TSC1–TSC2 complex, and the GTPase-activating protein (GAP) toward a small G protein referred to as the Ras homologue, which is enriched in the brain [48,49]. 

The suppression of mTORC1 mediated by REDD1 could downregulate the phosphorylation of p70S6K1 and 4E-BP1, which are associated with protein synthesis. In this study, ECR enhanced myotube length and width, which were decreased by treatment with rapamycin, a specific mTORC1 inhibitor, in DEX-induced muscle atrophic myotubes. GR binds to a glucocorticoid response element in the nucleus, alters chromatin confirmation, and activates target genes, such as ddit4, kif15, depdc6, sesn1, and mknk2, which function as the primary target genes for GR [50]. KLF15 is a member of the zinc-finger family of transcription factors, a GR-induced transcription factor related to the glucocorticoid–GR system and inhibits acetylation-mediated nuclear factor kappa B activation through the KLF15–p300 interaction [50]. 

Based on the results of the present study, we briefly examined the interaction between ellagic acid, one of the compounds in ECR, and muscle atrophy or sarcopenia using the STRING database together with biological processes, molecular functions, and cellular components via GO and KEGG pathways, which are frequently used in computational docking studies. There is no information about Cibotii Rhizoma or Cibot Rhizoma in Gene Expression Omnibus datasets managed by the National Center for Biotechnology Information, except data on the expression and phenotype from leaves and not the rhizome of “*Cibotium barometz*” (Platform Accession No. GPL23414; accessed 1 May 2023). Common targets between selected components of ECR detected by HPLC and muscle atrophy/sarcopenia were AKT1, as suggested partly by the present results, and CAV1. CAV1 is a scaffolding protein within caveolar membranes that functions also as a membrane adaptor to couple integrins to the tyrosine kinase Fyn and localizes to the Golgi apparatus and trans-Golgi-derived transport vesicles [51]. According to the annotation of public databases obtained in this study, CAV1 interacts directly with G-protein alpha subunits and can functionally regulate their activity; it is involved in the co-stimulatory signal essential for T cell receptor-mediated T cell activation and induces T cell proliferation and nuclear factor kappa B activation by binding to dipeptidyl peptidase 4. Furthermore, the results of the enrichment analysis showed that phosphatidylinositol-3,4-bisphosphate (Ptdins (3,4) P2) 5-kinase may be associated with this network. Ptdins (3,4,5) P3 -binding proteins tend to bind to Ptdins (3,4) P2-like proteins with a pleckstrin homology domain including TAPP1 or TAPP2, and Akt/PKB is the major protein kinase [52]. Considering that the prediction of the interaction between Cibotium Rhizome and muscle atrophy or sarcopenia was briefly performed in this study using computational analysis tools based on public databases, wide-ranging network research on Cibotium Rhizome will be needed in the future.

## 4. Materials and Methods

### 4.1. Reagents and Chemicals

Dulbecco’s modified Eagle’s medium (DMEM), 0.25% trypsin-ethylene diamine tetra acetic acid (EDTA), phosphate-buffered saline (PBS), and fetal bovine serum (FBS) were purchased from Welgene (Daegu, Republic of Korea). Horse serum was purchased from Gibco (Grand Island, NY, USA). Penicillin–streptomycin was purchased from Thermo Fisher Scientific (Waltham, MA, USA). Myosin heavy chain (MyHC), p-Akt, p-mTOR, MAFbx, MuRF1 and HSC70 antibodies were obtained from Santa Cruz Biotechnology (Santa Cruz, CA, USA). p-p70S6K and p-4E-BP1 antibodies were purchased from Cell Signaling Technology (Beverly, MA, USA). Horseradish peroxidase (HRP)-conjugated secondary antibodies were purchased from Jackson ImmunoResearch Laboratories (West Grove, PA, USA). Rapamycin was obtained from Calbiochem (St. Louis, MO, USA). Protocatechuic acid (analytical standard), (+)-catechin hydrate (≥98.0%, HPLC), p-coumaric acid (≥98.0%, HPLC), ellagic acid (≥95.0%, HPLC), chlorogenic acid (≥98.0%, titration), caffeic acid (≥98.0%, HPLC), dimethyl sulfoxide (DMSO), ethyl alcohol, formic acid, and DEX were purchased from Sigma-Aldrich Co. (St. Louis, MO, USA). Acetonitrile and methanol were purchased from Fisher Scientific Inc. (Cleveland, OH, USA), and ferulic acid was obtained from Toronto Research Chemicals (TRC, North York, ON, Canada).

### 4.2. Sample Preparation

The dried Cibotium Rhizome was purchased from Hanyakjae (Namyangju, Republic of Korea) in September 2021 and confirmed by Jaehun Hong (Samhonggungae Yakupsa, Republic of Korea). A voucher specimen (PC 2022-01) was deposited at the herbarium of the university for further reference. In total, 50 g of the dried part of the Cibotium Rhizome was extracted with 500 mL of 70% ethanol (1:10, *w*/*v*) at room temperature for 24h. Thereafter, it was filtered through a sterile cheesecloth layer (Daehanwejae, Choongjoo, Republic of Korea). The filtrates were evaporated by a rotary evaporation concentrator (N-1300, EYELA, Tokyo, Japan) at 40 °C, and the supernatants were lyophilized. The yield was 8.53% of the initial dry weight of the Cibotium Rhizome. The final extract was stored at −20 °C until use [22].

### 4.3. Compound Analysis of ECR by HPLC

The compounds of ethanol extracts of Cibotium Rhizome (ECR) were analyzed by high-performance liquid chromatography (HPLC) using a Waters 2695 system equipped with a Waters 2489 UV detector. All samples were used after dissolving in acetonitrile including 1% phosphoric acid, filtering with a 0.45 µm membrane filter (Millipore, Billerica, MA, USA), and were separated on an Atlantis C18 column (150 × 4.6 mm, 5 µm, Waters Co., Milford, MA, USA) at 34 °C. The mobile phase consisted of 1% phosphoric acid (solvent A) and 100% acetonitrile (solvent B) with a 1.0 mL/min flow rate. Gradient elution was performed with time-regulated cycles as follows: 0 min, 90% solvent A and 10% solvent B; 0–27 min, 60% solvent A and 40% solvent B; 28–55 min, 56% solvent A and 44% solvent B; 56–60 min, 90% solvent A and 10% solvent B. All samples were passed through membrane filters with a 0.45 µm pore size, and 10 µL of each filtered sample was injected into an HPLC device. Absorbance was monitored at 280 nm for protocatechuic acid, (+)-catechin hydrate, *p*-coumaric acid, and ellagic acid, and at 320 nm for chlorogenic acid, caffeic acid, and ferulic acid. The results were quantified using a curve corresponding to standard materials and are presented in micrograms per gram [22]. The quantitative curve equation for each standard is shown in Supplementary Table S1.

### 4.4. Cell Culture and Differentiation

C2C12 cells (passages 4–17, CRL-1772; ATCC, Manassas, VA, USA) were incubated at 37 °C in a 5% CO_2_ atmosphere. The C2C12 cells were cultured in DMEM with 4500 mg/L D-glucose, 584 mg/L L-glutamine, 110 mg/L sodium pyruvate, 1200 mg/mL sodium bicarbonate, 100 Units/mL penicillin, 100 µg/mL streptomycin, and 10% FBS. For C2C12 differentiation, 9 × 10^4^ cells were seeded and then, cells at 70–80% confluence were replaced with the differentiation medium containing DMEM supplemented with 2% horse serum for 6 days. The C2C12 cells were treated with ECR (50 and 100 µg/mL) and DEX (39.2 µg/mL) for 24 h. The assessments of ECR on cell viability were performed using 3-(4,5-dimethylthiazol-2-yl) (MTT; Duchefa Biochemie, Haarlem, The Netherlands) as previously described [53].

### 4.5. Jenner–Giemsa Staining

As described previously in the Methods, C2C12 myotubes were washed with PBS, fixed with 100% methanol for 5 min, and left to air dry for 10 min [54]. Jenner’s staining solution (EMS, Hatfield, PA, USA) was diluted 1:3 in PBS, and 1 mL was incubated in the wells for 5 min, followed by washing with distilled water. The wells were then incubated with 1 mL Giemsa stain (Sigma Aldrich, St. Louis, MO, USA) diluted 1:20 in distilled water for 10 min at room temperature, and then washed again with distilled water. Five randomly selected regions from each well were photographed using a fluorescence microscope (Eclipse TS2-LS, Nikon, Tokyo, Japan). The width and length of the myotubes were measured and quantified using the Nikon analysis software version 5.30.03 64-bit (NIS-Element D, Basic Research Software, Nikon, Tokyo, Japan), and myotube density was measured and quantified using the free image processing software ImageJ (ImageJ bundled with 64-bit Java 8, National Institutes of Health, Bethesda, MA, USA).

### 4.6. Western Blot Analysis

C2C12 myotubes were lysed using a cold lysis buffer (100 mM Tris-HCl, 100 mM NaCl, 0.5% Triton-X) containing 1 mM sodium orthovanadate, 10 mM sodium fluoride, and protease inhibitor cocktail (GenDEPOT, Barker, TX, USA). Whole cell lysates were centrifuged at 13,000 rpm for 10 min at 4 °C, and the supernatant was transferred into new tubes. The protein concentration was measured using the Bradford assay. Equal amounts of each sample protein were separated on a sodium dodecyl sulfate polyacrylamide gel and transferred to a polyvinylidene difluoride membrane. The membranes were blocked with 5% skim milk and incubated successively with primary antibodies against MyHC (1:1000 dilution), p-Akt (1:500 dilution), p-mTOR (1:500 dilution), p-p70S6K (1:1000 dilution), p-4E-BP1 (1:1000 dilution), MAFbx (1:1000 dilution), MuRF1 (1:1000 dilution), and HSC70 (1:2000 dilution) at 4 °C overnight. The membranes were washed with Tris-buffered saline-Tween 20 (10 mM Tris-HCl, 150 mM NaCl, and 0.05% Tween-20), and then incubated with HRP-conjugated mouse or rabbit secondary antibodies for 2 h at room temperature. Reactive bands were visualized using the ChemiDoc System (300; Azure Biosystems, Dublin, CA, USA), and specific band intensities were quantified using the software ImageJ (ImageJ bundled with 64-bit Java 8) [53].

### 4.7. Quantitative Real-Time-PCR

Total RNA was isolated using RNAiso Plus (TAKARA, Kyoto, Japan) [22]. The concentration of the extracted RNA was measured at 260 nm and 280 nm using a spectrophotometer (UL 61010-1, Thermo Fisher Scientific), and only RNA having an A260/A280 ratio of 1.8 to 2.0 was used. cDNA was synthesized using the AMPIGENE^®^ cDNA Synthesis Kit (Enzo Life Sciences, Farmingdale, NY, USA) according to the manufacturer’s protocol. Quantitative real-time PCR analysis was performed using a 20 µL reaction mixture using AMPIGENE^®^ qPCR Green Mix Hi-ROX (Enzo Life Sciences, Farmingdale, NY, USA). The results were calculated using the 2^−ΔΔCt^ analysis method and normalized to the 18s rRNA gene level. Primer sequences for the PCR of the two target genes, KLF15 and REDD1, and the internal reference gene, 18s RNA, are listed in Table 3.

### 4.8. Immunofluorescence Staining

C2C12 myotubes were differentiated in 6-well plates. Myotubes were fixed with 3% paraformaldehyde for 15 min, permeabilized with 0.1% saponin, blocked with 1% bovine serum albumin in PBS, and then stained with total MyHC antibody (1:500 dilution) overnight at 4 °C. Moreover, the myotubes were rinsed with PBS, incubated for 60 min at room temperature with Alexa Fluor 488-conjugated anti-mouse IgG (1:1000 dilution; Cell Signaling Technology, Beverly, MA, USA), and incubated for 5 min at room temperature with 1 µg/mL 4′,6-diamidino-2-phenylindole (DAPI; Roche, CA, USA). Images were captured and analyzed using a Nikon Eclipse TS2-LS microscope. The number of nuclei per myotube and a total number of nuclei were determined [55].

### 4.9. Computational Database Analysis

Among compounds of ECR confirmed by HPLC in the present study, we investigated compounds with DL ≥ 0.18, OB ≥ 30%, and Caco-2 ≥ −0.4 according to absorption, distribution, metabolism, and extraction using a traditional Chinese medicine systems pharmacology database. Targets for active ingredients selected in ECR were retrieved from the search tool for interactions of chemicals (STITCH) version 5.0 with an interaction score ≥ 0.7. Targets that have already been identified in relation to sarcopenia/skeletal muscle atrophy were obtained from the GeneCards human gene database. All organisms used for the computational analysis in the present study were limited to *Homo Sapiens*. Through a Venn diagram, overlapped targets were identified. In addition, we utilized the STRING 11.5 database together with biological processes, molecular functions, and cellular components via the Gene Ontology (GO) and Kyoto Encyclopedia of Genes and Genomes (KEGG) pathways, which are frequently used for computational docking studies.

### 4.10. Data Analysis

All data were expressed as the mean ± standard deviation (S.D) and analyzed by SPSS 25 Statistics (IBM, Chicago, IL, USA). The statistical significance of differences between mean values was assessed using Duncan’s multiple range test, Dunnett’s *t*-test, and an independent *t*-test. Enrichment analyses were performed using Metascape platform. Statistical significance was set at *p* < 0.05. All experiments were repeated at least three times to ensure reproducibility.

## 5. Conclusions

To our knowledge, this study is the first study to demonstrate that ECR alleviates DEX-induced muscle atrophy by repressing REDD1 and KLF15 expression in C2C12 myotubes. Cibotium Rhizome has been used as a folk remedy for the treatment of bone or joint diseases in Asia; however, to date, there have been no studies investigating its recovery effects on muscle atrophy in C2C12 myotubes. Although the mechanisms of sarcopenia or skeletal muscle atrophy have not yet been completely elucidated, the results of this study may provide fundamental data for the development of preventive or therapeutic agents originating from natural products without side effects. In addition, we analyzed the interaction between components, targets, and disease pathways using public database tools based on our HPLC analysis; however additional molecular docking studies should be performed with a well-planned experimental design in the future.

In this study, we investigated whether ECR regulates anabolic and catabolic processes associated with protein synthesis and degradation in DEX-induced muscle atrophic myotubes. ECR increased the width and length of myotubes and MyHC expression and enhanced the phosphorylation of Akt, mTOR, p70S6K, and 4E-BP1, whereas it attenuated the protein expression of MAFbx, MuRF1 and mRNA expression of REDD1 and KLF15 upregulated by DEX. In conclusion, ECR is a potential preventive or therapeutic agent that alleviates muscle atrophy caused by various stress inducers and diseases such as sarcopenia.

## Figures and Tables

**Table 1:** The main compounds of ethanol extracts of Cibotium Rhizome (ECR).

Compounds (CAS Number)	2-Dimensional Chemical Structure	3-Dimensional Chemical Structure	Retention Time (min)	Quantity (µg/g Extract) [22]	Classification	Absorption (nm)	DL OB (%) Caco-2	Reference
Protocatechuic acid (99-50-3)	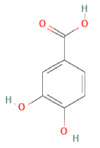	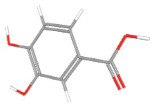	8.724	562.28 ± 0.77	Benzoates	280	0.04 25.37 0.10	[23]
(+)-Catechin hydrate (225937-10-0)	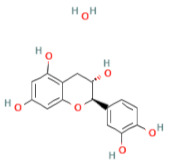	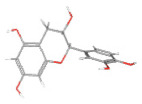	11.744	2233.07 ± 1.56	Catechins	280	0.24 17.83 0.10	[24]
*p*-Coumaric acid (501-98-4)	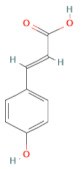	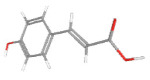	20.995	166.90 ±52.64	Monolignols	280	0.04 43.29 0.46	[25]
Ellagic acid (476-66-4)	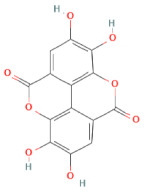	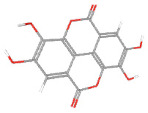	24.152	274.78 ± 1.48	Tannins and Galloyl derivatives	280	0.43 43.06 −0.44	[26]
Chlorogenic acid (327-97-9)	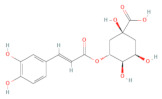	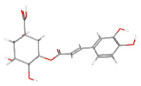	10.950	5068.92 ± 6.20	Monolignols	320	0.31 13.61 −1.33	[27]
Caffeic acid (331-39-5)	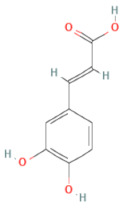	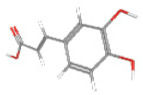	14.309	313.30 ± 65.53	Monolignols	320	0.05 54.97 0.27	[23]
Ferulic acid (1135-24-6)	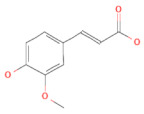	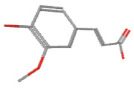	24.148	874.05 ± 1.20	Monolignols	320	0.06 40.43 0.96	[23]

**Table 2:** Functional enrichments in network.

	Count	P. Adjust Value
Biologic process		
TORC2 signaling	3	0.00028
Mast cell differentiation	2	0.0075
Natural killer cell chemotaxis	2	0.0075
Caveola assembly	2	0.0104
Receptor-mediated endocytosis of virus by host cell	2	0.0104
Negative regulation of long-chain fatty acid import across plasma membrane	2	0.0104
Myeloid progenitor cell differentiation	2	0.0104
Postive regulation of neutrophil apoptotic process	2	0.0133
Establishment or maintenance of actin cytoskeleton polarity	2	0.0133
Mast cell chemotaxis	2	0.0133
Molecular function		
Phosphatidylinositol-3,4-bisphosphate 5-kinase activity	4	1.56 × 10^−5^
Phosphatidylinositol-4,5-bisphosphate 3-kinase activity	4	1.95 × 10^−5^
1-phosphatidylinositol-4-phosphate 3-kinase activity	4	1.95 × 10^−5^
1-phosphatidylinositol-3-kinase activity	4	4.60 × 10^−5^
Phosphatidylinositol-3,4-trisphosphate binding	4	0.0022
Protein serine/threonine kinase activator activity	4	0.0026
Insulin receptor binding	3	0.0120
Phosphatidylinositol-3,4-bisphosphate binding	3	0.0165
Chitin binding	2	0.0431
Guanylate kinase activity	2	0.0498
Cellular component		
TOR complex	5	1.33 × 10^−6^
Phosphatidylinositol-3-kinase complex	5	2.93 × 10^−5^
Extrinsic component of membrane	10	2.93 × 10^−5^
TORC2 complex	4	2.99 × 10^−5^
Caveolar macromolecular signaling complex	2	0.0087
Mast cell granule	3	0.0090
Cytoplasmic side of plasma membrane	5	0.0202
Phosphatidylinositol-3-kinase complex, class1	2	0.0207
TORC1 complex	2	0.0207
Lamellipodium	5	0.0448
KEGG pathways		
Longevity regulating pathway-multiple	10	2.99 × 10^−12^
Acute myeloid leukemia	10	4.88 × 10^−12^
Longevity regulating pathway	10	4.34 × 10^−11^
Central carbon metabolism in cancer	9	1.99 × 10^−10^
Regulation of lipolysis in adipocytes	8	1.15 × 10^−9^
Endometrial cancer	8	1.53 × 10^−9^
Non-small cell lung cancer	8	3.98 × 10^−9^
Carbohydrate digestion and absorption	7	7.12 × 10^−9^
VEGF signaling pathway	7	2.76 × 10^−8^
GnRH secretion	7	4.42 × 10^−8^

**Table 3:** Primer sequences used for real-time RT-PCR.

Target Gene	Primer Sequences (5′→3′)	NCBI Accession Number
KLF15	F: TGGTACCATCCTCCAACTTGAA R: CAATAGGTTTGGCGGCAATG	NM_023184.4
REDD1	F: CCTAGCCTCTGGGATCGTTTC R: ATCAGCGGCCGGAGTTC	NM_029083.2
18s rRNA	F: AACCCGTTGAACCCCATT R: CCATCCAATCGGTAGTAGCG	NR_003278.3

## Data Availability

Not applicable.

## Supplementary Materials

The supporting information can be downloaded at: https://www.mdpi.com/article/10.3390/ijms241914798/s1.
