# Supplementary material for: Protective Role of Ethanol Extract of Cibotium barometz (Cibotium Rhizome) against Dexamethasone-Induced Muscle Atrophy in C2C12 Myotubes
Source: Int J Mol Sci. 2023 Sep 30;24(19):14798. doi: 10.3390/ijms241914798 (PMC10573348; doi:10.3390/ijms241914798)
Supplement: Supplementary file 1 [file ijms-24-14798-s001.zip › ijms-2561372-supplementary.pdf]

Supplementary Table S1. The quantitative curve equation for each standard.

	**Equation (R^2)**
Protocatechuic acid	y = 16.2422 x-2.8709 (0.9997)
(+)-Catechin hydrate	y = 8.4265 x-22.0923 (0.9915)
p-Coumaric acid	y = 44.2282 x-15.7941 (0.9998)
Ellagic acid	y = 8.1854 x-17.7510 (0.9971)
Chlorogenic acid	y = 17.1286 x-16.3361 (0.9993)
Caffeic acid	y = 35.3910 x-7.5743 (0.9999)
Ferulic acid	y = 39.3611 x-11.9731 (0.9999)
